# Presentation and Outcomes of CNS Tumors Associated With Phakomatoses Syndromes From a Specialized Neuro‐Oncology Practice in India

**DOI:** 10.1002/cam4.71483

**Published:** 2026-02-04

**Authors:** Anuradha Krishnan, Yamini Baviskar, Abhishek Chatterjee, Archya Dasgupta, Sridhar Epari, Arpita Sahu, Girish Chinnaswamy, Nandini Menon, Aliasgar Moiyadi, Tejpal Gupta, Jayant Sastri‐Goda

**Affiliations:** ^1^ Department of Radiation Oncology Tata Memorial Centre, Homi Bhabha National Institute Mumbai India; ^2^ Department of Pathology Tata Memorial Centre, Homi Bhabha National Institute Mumbai India; ^3^ Department of Radiology Tata Memorial Centre, Homi Bhabha National Institute Mumbai India; ^4^ Department of Paediatric Oncology Tata Memorial Centre, Homi Bhabha National Institute Mumbai India; ^5^ Department of Medical Oncology Tata Memorial Centre, Homi Bhabha National Institute Mumbai India; ^6^ Department of Surgical Oncology Tata Memorial Centre, Homi Bhabha National Institute Mumbai India

**Keywords:** neurofibromatosis, phakomatoses, primary central nervous system tumors

## Abstract

**Purpose:**

Phakomatoses‐associated primary central nervous system (CNS) tumors are therapeutically challenging due to young age of onset, multiple tumors, and prolonged morbidity from long‐term survival. This study evaluated demographics, survival, and prognostic factors of patients with phakomatoses‐associated CNS tumors treated at a specialized neuro‐oncology service in India.

**Materials and Methods:**

Consecutive patients diagnosed and managed between 2000 and 2022 were included in this retrospective study. Data were retrieved from electronic medical records. Treatment decisions were multidisciplinary and included maximal safe resection, radiation (RT), and systemic therapy as indicated. Kaplan–Meier survival analysis evaluated overall survival (OS) and progression‐free survival (PFS). Univariate analyses used the log‐rank test, and multivariate analyses the restricted mean survival time.

**Results:**

A total of 121 patients were analysed: NF1 (61.2%), NF2 (23.1%), VHL (10.7%), and TSC (5.0%). For NF1, median follow‐up was 36.5 months (95% CI: 1–254); 3‐year PFS and OS were 76.4% (95% CI: 66.5–87.8) and 87.7% (95% CI: 80.1–96.1) respectively. On univariate analysis, NF1 high‐grade glioma patients who did not receive RT had inferior outcomes, though not significant on RMST. For NF2, median follow‐up was 40.5 months (95% CI: 1–199); 5‐year PFS and OS were 37.3% (95% CI: 20.5–68.1) and 100% respectively. For VHL, median follow‐up was 66.5 months (95% CI: 3–426); 5‐year PFS and OS were 77.9% (95% CI: 54.6–100) and 100% respectively. For TSC, median follow‐up was 71 months (95% CI: 1–228); 5‐year PFS and OS were 75% (95% CI: 42.5–100) and 100% respectively.

**Conclusion:**

Phakomatoses‐associated CNS tumors show promising outcomes with multimodality management. Further research into targeted multimodality treatment is warranted.

## Graphical abstract

Encouraging survival is achieved in phakomatosis syndromes with conventional multimodality treatment (Surgery, RT, chemotherapy). There is an urgent need for easily administered systemic therapies which improve both quality and quantity of life.

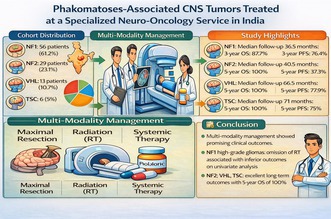

## 1 Introduction

The phakomatoses syndromes collectively refer to a group of diseases with multi‐systemic involvement that primarily affect structures of ectodermal origin (skin, eyes, nervous system). The common phakomatoses constitute Neurofibromatosis (NF) types 1 (von‐Recklinghausen's disease) and 2, von‐Hippel‐Lindau disease (VHL) and Tuberous sclerosis (TS), with continuously evolving diagnostic criteria with accumulating clinical and genetic knowledge [1, 2, 3, 4]. Tumors form a common association in phakomatoses and can present across multiple organ systems, especially in the central nervous system (CNS), with a wide spectrum ranging from benign to highly malignant entities. Treating these tumors is challenging due to the young age at presentation, multiplicity in tumor sites and numbers, associated significant morbidity and sensitivity of underlying tissues to treatment modalities, especially radiation [5]. Additionally, the long‐term survival observed renders patients deeply susceptible to treatment‐induced long‐term morbidity. Treatment in the form of local maximal safe resection and RT has been typically reserved for progressive or symptomatic disease and provides modest benefits. Systemic therapies (holding the inherent advantage of acting at multiple sites of disease) have been recently developed and have shown promising results, but are limited in terms of availability, cost, and response [6, 7, 8]. There is a paucity of real‐world data on treatment outcomes of brain tumors in patients with phakomatoses, which will provide useful benchmark data to design future clinical trials implementing novel therapeutics and treatment strategies. The current study summarizes the clinico‐pathological spectrum and outcomes attained with multimodality therapy in patients with phakomatoses syndrome‐associated brain tumors treated in a specialized neuro‐oncology practice in India.

## 2 Materials and Methods

### 2.1 Institutional Ethics Approval

The current study was approved by the Institutional Review Board (IRB), which functions in accordance with the Declaration of Helsinki. Given less than minimal risk to participants, being a retrospective observational study, a waiver of consent was sought and duly obtained

### 2.2 Patient Population

The records of patients diagnosed with phakomatoses syndromes (NF1, NF2, VHL, TSC) satisfying clinical diagnostic criteria who were registered between 2000 and 2022 were identified from a prospectively maintained neuro‐oncology database. Demographic and clinicopathological data, treatment details, and outcomes were retrieved retrospectively from hospital case files and electronic medical records.

### 2.3 Treatment

Decision‐making processes typically involve Multidisciplinary Joint Clinics (MDJCs) followed by shared decision‐making with the patient and caregivers. Pre‐treatment evaluation necessarily included plain and contrast magnetic resonance imaging (MRI) of the brain and spine with relevant axial sections through areas of interest in the spine. Following evaluation in an MDJC, patients were typically treated for symptomatic disease or progressive disease. Patients underwent maximal safe resection whenever feasible. Radiotherapy was typically reserved for symptomatic progressive or residual disease or gross disease in areas not amenable to surgery. For low‐grade histology, RT doses were 50–54 Gy in 30 fractions, whereas for high‐grade histology, typically 55.8–59.4Gy in 31–33 fractions was delivered. Concurrent and adjuvant chemotherapy typically consisted of oral temozolomide (TMZ). Systemic chemotherapy typically consisted of Vinblastine (VBL) based monotherapy in NF1 patients and Bevacizumab in NF2 patients. Tuberous sclerosis patients were considered for MTOR inhibition with everolimus. For high‐grade histology, patients were assessed with MRI after 1 month post‐RT to assess response. Patients were followed up 3‐monthly following the initial response assessment till 2 years and 6 monthly thereafter for 5 years. For low‐grade histology, patients were assessed with MRI 8–12 weeks post‐initial treatment (surgery, chemotherapy, or RT) to assess response. Patients were followed up 6‐monthly following the initial response assessment till 5 years. Standard imaging assessment as per established criteria was performed (RANO, BTRADS). Further imaging was planned for symptomatic worsening or on suspicion of progression at the discretion of the treating physician.

### 2.4 Outcome Measures

Progression‐free survival (PFS) and overall survival (OS) were evaluated as the main outcome measures of efficacy. PFS was defined as the time interval between diagnosis and documented clinico‐radiological progression or death, while OS was calculated from the date of diagnosis to death from any cause.

### 2.5 Statistical Analysis Plan

Clinicopathological and demographic variables were summarized using descriptive statistics and analyzed using measures of central tendency and dispersion. Continuous variables were summarized using numbers, median, and interquartile range (IQR), while categorical variables were summarized using numbers and percentages. Time‐to‐event outcomes (PFS and OS) were calculated using the product limit method of Kaplan–Meier and reported as point estimates with corresponding 95% confidence interval (CI). The statistical plan included univariate analysis of prognostic variables using the Log‐Rank test after dichotomizing variables of known or deemed prognostic significance. The statistical plan included multivariate Cox Regression analysis in the event of significant prognostic factors (defined as *p* ≤ 0.10) emerging on univariate analysis, which displayed non‐collinearity and did not violate proportional hazards assumptions on testing with scaled Schoenfeld residuals. In the event of such non‐proportional hazards emerging, a Restricted Mean Survival Time (RMST) analysis was planned to test the putative significance of factors affecting outcome measures (OS and PFS). Analysis was performed using Statistical Package for Social Services (SPSS) Version 24.0 (IBM Corp., Armonk, IL, U.S.A) and R Studio version 4.02 (CRAN, R Project Vienna).

## 3 Results

A total of 121 patients from a prospectively maintained institutional database with CNS tumors fulfilling contemporary clinico‐radiological diagnostic criteria of neuro‐cutaneous syndromes from 2000 to 2022 were included in the study. Seventy‐four patients (61.2%) were diagnosed with NF1, 28 patients (23.1%) with NF2, 13 patients (10.7%) with VHL, and 6 patients (5.0%) with TSC complex.

Baseline characteristics of the entire study cohort (*N* = 121) are summarized in Table 1. A mild male gender predilection was observed in our cohort {*n* = 68 (56%)} along with a bimodal age distribution with the first peak below 12 years of age (children) (45 patients, 36.6%) and the second between 19 and 35 years of age (young adults) (39 patients, 31.7%). The median age of the cohort was 18 years with an inter‐quartile range (IQR) of 9–29 years.

**TABLE 1:** Cohort characteristics.

	NF1 (*n* = 74)	NF2 (*n* = 28)	TS (*n* = 6)	VHL (*n* = 13)
Demographics				
Age ≤ 16 years	45 (60.8%)	20 (71.4%)	5 (83.3%)	1 (7.7%)
Age > 16 years	29 (39.2%)	8 (28.6%)	1 (16.7%)	12 (92.3%)
Male	43 (58.1%)	14 (50.0%)	3 (50.0%)	7 (53.8%)
Female	31 (41.9%)	14 (50.0%)	3 (50.0%)	6 (46.2%)
Baseline imaging				
CT	1 (1.4%)	2 (7.1%)	1 (16.7%)	—
MRI	73 (98.6%)	26 (92.9%)	5 (83.3%)	13 (100%)
Tumor grade				
High grade	23 (31.1%)	—	1 (16.7%)	0
Low grade	51 (68.9%)	28 (100%)	5 (83.3%)	13 (100%)
Syndrome‐specific stigmata				
CALM	62 (83.8%)	6 (21.4%)	—	—
Axillary freckling	6 (8.1%)	1 (3.6%)	—	—
Neurofibroma	37 (47.3%)	10 (35.7%)	1 (16.7%)	—
Lisch nodules	28 (37.8%)	—	—	—
PNST	—	5 (17.9%)	—	—
Pancreatic cyst	—	—	—	5 (38.5%)
Hamartoma	1 (1.4%)	—	—	—

*Note:* Percentages are calculated within each syndrome group. Blank cells indicate no cases observed.

Abbreviations: CALM, café‐au‐lait macules; CT, computed tomography; MRI, magnetic resonance imaging; NF1, neurofibromatosis type 1; NF2, neurofibromatosis type 2; PNST, peripheral nerve sheath tumor; TS, tuberous sclerosis; VHL, Von Hippel Lindau.

Individual entities and outcomes are discussed in the subsequent sections.

### 3.1 Neurofibromatosis 1 (NF1)

Seventy‐four patients met the diagnostic criteria for NF 1 [2]. Of the 74 patients, high‐grade tumors (Grades III and IV) were seen in 23 patients (31.1%), and low‐grade tumors (Grades I and II) were seen in 51 patients (68.9%). The most common symptom in patients with NF1 was headache, followed by diminution of vision and protrusion of the eyeball owing to optic pathway glioma. Phenotypic manifestations were seen in all patients, with Café Au Lait Macules (CALMs) being the commonest finding seen in 62 patients (84%). Neurofibromas, axillary freckling, and ocular findings (Lisch nodules and/or choroidal abnormalities) were seen in 37 (47.3%), 6 (8.1%), and 28 (37.8%) patients, respectively.

The most common tumor in patients with NF1 was Optic Pathway Glioma (OPG), seen in 24 (32.4%) patients, which most commonly involved the optic nerves (18 patients, unilateral—8, bilateral—6), followed by chiasmatic gliomas (10 patients). Brainstem, tectal plate, cerebellar, and supratentorial low‐grade gliomas were seen in 21 (28.3%) patients, of which histopathological diagnosis was available in 10 patients, with the majority (8 patients) being pilocytic astrocytomas.

High‐grade glioma was seen in 23 patients (31.1%), of which 18 (24.3%) patients had Glioblastoma {(GBM)WHO grade IV} on histology, 2 (2.7%) patients had anaplastic astrocytoma (WHO grade III), while 2 patients (2.7%) did not have a histopathological diagnosis although MR imaging features were suggestive of a high‐grade neoplasm. Other miscellaneous CNS tumors seen were pleomorphic xanthoastrocytoma (2 patients, 2.7%), malignant peripheral nerve sheath tumors (2 spinal and 1 cerebellar, 4%), meningioma (1 patient, 1.3%), and spinal neurofibromas.

The majority of optic pathway gliomas did not undergo any biopsy and received low‐grade glioma vinblastine monotherapy for at least 6–12 months (68.6%), while the high‐grade gliomas underwent maximal safe resection (91.3%) followed by RT in 74% of patients. The treatment details of all the patients have been summarized in Table 2.

**TABLE 2:** Treatment details, patterns of relapse and patient status.

Variable	NF1—high grade (*n* = 23)	NF1—low grade (*n* = 51)	NF2 (*n* = 28)	TS (*n* = 6)	VHL (*n* = 13)
Surgery					
Yes	21 (91.3%)	16 (31.4%)	21 (75.0%)	4 (66.7%)	12 (92.3%)
No	2 (8.7%)	35 (68.6%)	7 (25.0%)	2 (33.3%)	1 (7.7%)
Extent of resection (EOR)					
GTR/NTR	13 (62.0%)	10 (62.5%)	16 (57.1%)	3 (50.0%)	12 (100%)
STR/Biopsy	8 (38.0%)	6 (37.5%)	5 (17.9%)	1 (16.7%)	0
External beam radiotherapy (EBRT)					
Yes	17 (74.0%)	10 (19.6%)	14 (50.0%)	1 (16.7%)	0
No	6 (26.0%)	41 (80.4%)	14 (50.0%)	5 (83.3%)	13 (100%)
Adjuvant treatment					
Temozolomide	9 (39.1%)	0	0	1 (16.7%)	0
Bevacizumab	0	0	1 (3.6%)	0	0
Imatinib	0	0	0	1 (16.7%)	0
Patterns of relapse					
No recurrence	10 (43.5%)	40 (78.4%)	11 (39.3%)	4 (66.7%)	7 (53.8%)
Local	11 (47.8%)	9 (17.6%)	15 (53.6%)	2 (33.3%)	3 (23.1%)
Distant	0	2 (4.0%)	2 (7.1%)	0	3 (23.1%)
Local + distant	2 (8.7%)	0	0	0	0
Status at last follow‐up					
Alive, NED	7 (30.4%)	16 (31.4%)	12 (42.9%)	4 (66.7%)	6 (46.2%)
Alive with disease	8 (34.8%)	33 (64.6%)	15 (53.6%)	2 (33.3%)	7 (53.8%)
Died due to disease	8 (34.8%)	1 (2.0%)	1 (3.6%)	0	0
Died due to other cause	0	1 (2.0%)	0	0	0

*Note:* Percentages are calculated within each subgroup.

Abbreviations: EBRT, external beam radiotherapy; GTR, gross total resection; NED, no evidence of disease; NF1, Neurofibromatosis type 1; NF2, Neurofibromatosis type 2; NTR, near total resection; STR, subtotal resection; TS, tuberous sclerosis; VHL, Von Hippel Lindau.

The median follow‐up for the NF1 cohort was 36.5 months (95% CI: 1–254 months), with 3‐year PFS and OS of 76.4% (95% CI: 66.5%–87.8%) and 87.7% (95% CI: 80.1%–96.1%), respectively. For low‐grade gliomas, the 3‐year PFS and OS were 89.7% (95% CI: 80.5%–100%) and 100%, while for high‐grade gliomas, it was 47.7% (95% CI: 30.5%–74.6%) and 60.6% (95% CI: 42.6%–82.3%), respectively (Table 3).

**TABLE 3:** Comparative studies of patients of phakomatoses with primary Central Nervous System (CNS) tumors.

Author, year	Type of study	Cohort characteristics	Endpoints	Results
*Neurofibromatosis‐1 (NF1)*				
Merchant et al. [9]	Phase II study	*N* = 78 pediatric patients with LGG, 13 patients had NF1	Estimation of disease control with 10 mm CTV margin in conformal RT treatment	Patients with NF1had higher rates of baseline vasculopathy that worsened after therapy, 5 mm margins were adequate
Wentworth et al. [10]	Retrospective study	*N* = 18 NF1 patients with 18 tumors treated with RT 16%—acoustic neuroma 6%–ependymoma 11%—LGG 60%—meningioma 17%—VS	PFS and OS 5‐year OS—94% 5‐year PFS—75% for acoustic neuroma 100% for ependymoma 75% for LGG 86% for meningioma 100% for VS And 50% hearing preservation rate	RT should be considered in NF patients with progression on imaging in CNS tumors RT provides good OS and PFS rates
Huttner et al. [11]	Retrospective review	*N* = 5 NF1 patients with GBM 56 non NF1 patients with GBM	Survival and factors affecting survival	60% 2‐year OS with NF1 25% 2‐year OS with non NF1
Ater et al. [12]	Non randomized comparison of NF1 vs. non‐NF1 patients with progressive LGG	*N* = 127 NF1 patients treated with Carboplatin‐Vincristine (CV)	69% 5‐year EFS for CV‐NF1 39% for CV‐nonNF1	NF1 patients had better outcomes than non‐NF1 patients 3 SMNs at a median of 7.8 years in NF1 vs. none in non‐NF1 patients
Tsang et al. [13]	Retrospective study	*N* = 89 14—NF1 patients with OPG treated with chemotherapy and/or RT	EFS and OS Prognostic factors affecting survival	10‐year EFS with NFI—61.9% vs 67.5% without NF1 10‐year OS with NF1—92.3% vs 98.4% with non NF1 4/14 (28.5%) patients with NF1 developed second neoplasm
Spyris et al. [14]	Case series	*N* = 5 3—HGG 1—Anaplastic PXA 1—LGG	To facilitate appropriate treatment for HGG in NF1 patients	NF1 patients with HGG have better prognoses compared to non‐ NF1 counterparts
TMH study, 2025	Retrospective study	*N* = 74 23—HGG 51—LGG	OS and PFS Median f/u—36.5 months	HGG 3‐year PFS—47.7% 3‐year OS—60.6% LGG 3‐year PFS—89.7% 3‐year OS—100%
*Neurofibromatosis‐2 (NF2)*				
Plotkin et al. [15]	Retrospective study	*N* = 55 NF2 patients with spinal ependymoma 20% had Sx for symptomatic progression	Median diagnosis from diagnosis till identification of ependymomas was 5 years Review of clinical and treatment details	NF2‐related ependymomas have an indolent growth Surveillance is reasonable for asymptomatic Surgical resection for symptomatic and resectable
Farschtschi et al. [18] 2016	Retrospective study	*N* = 8 NF2 patients Symptomatic spinal ependymoma treated with Bev (3.75–7.5 mg/kg)	Clinical and radiographic (> 20% reduction) response	All patients had clinical benefit; half the patients had radiographic response at 3–6 months. No pseudo‐response
Champeaux‐Depond et al. [16]	Retrospective study	*N* = 184 patients operated on for 315 meningiomas 30%—also received SRS/RT	OS Factors affecting OS	10‐year OS—73.2% Malignant meningioma predictor of worse OS
Fujii et al. [17]	BeatNF2 trial, Randomized, double blind, placebo‐controlled phase III trial	NF2 associated VS—randomized to Bev (5 mg/kg) for 46 weeks vs. placebo for 22 weeks f/b Bev for 22–46 weeks	Improved hearing at 22 weeks	Ongoing study
Mohammed et al. [18]	Retrospective study	*N* = 204, NF2 associated meningiomas in 39 patients treated with Gamma knife SRS	Demographic details and outcomes studied	High RT dose and low number of meningiomas at presentation: 10‐year PFS—94.8% 10% radiation‐induced side effects No malignant transformation
Evans et al. [19]	Retrospective audit	*N* = 266, NF2 patients with VS, meningioma and spinal tumors treated with RT as first line treatment	Subsequent rates of CNS Malignancy/Malignant Progression (M/MP) and survival rates	6% CNS M/MP in patients treated with RT 30‐year survival, RT—45.6% vs. Control—66.4%, *p* = 0.02
Shrivastava et al. [20]	Retrospective study	*N* = 81 NF2 patients with meningiomas 98%—SRS 2%—fractionated RT	Growth control Treatment control 3. Loss of serviceable hearing	80% growth control 74% treatment control 10 year OS: 71% 10‐year preservation of serviceable hearing: 53% No second malignancy in 10 years
TMH study, 2025	Retrospective study	*N* = 28 Meningioma Schwannoma Ependymoma	OS and PFS Median f/u—40.5 months	Schwannoma 5‐year PFS—37.3% 5‐year OS—100% All patients of ependymoma and meningioma alive
*Von Hippel Lindau (VHL)*				
Binderup et al. [3]	Guidelines for diagnosis and surveillance	Annual neurological examination ± focused imaging and examination based on symptoms	From 15 years of age, MRI to be done once every 2 years	CNS Hemangioblastoma—surgical resection and Belzutifan
TMH study, 2025	Retrospective study	*N* = 13 Hemangioblastoma	OS and PFS Median f/u—66.5 months	5‐year PFS—77.9% 5‐year OS—100%
*Tuberous sclerosis (TS)*				
Northrup et al. [4]	International diagnostic, surveillance and management recommendations	MRI Brain to assess for the presence of tubers, SEN, migrational defects, and SEGA	One‐to‐three‐year interval surveillance MRI to be done	SEGA—maximal safe resection and mTOR inhibitors
TMH study, 2025	Retrospective study	*N* = 6 SEGA	OS and PFS Median f/u—71 months	5‐year PFS—75% 5‐year OS—100%

Abbreviations: EFS, event free survival; f/u, follow‐up; GBM, glioblastoma; HGG, high‐grade glioma; LGG, low‐grade glioma; MRI, magnetic resonance imaging; OPG, optic pathway glioma; OS, overall survival; PFS, progression free survival; RT, radiotherapy; SEGA, subependymal giant cell astrocytoma; SEN, subependymal nodules; VS, vestibular schwanomma.

**FIGURE 1:**
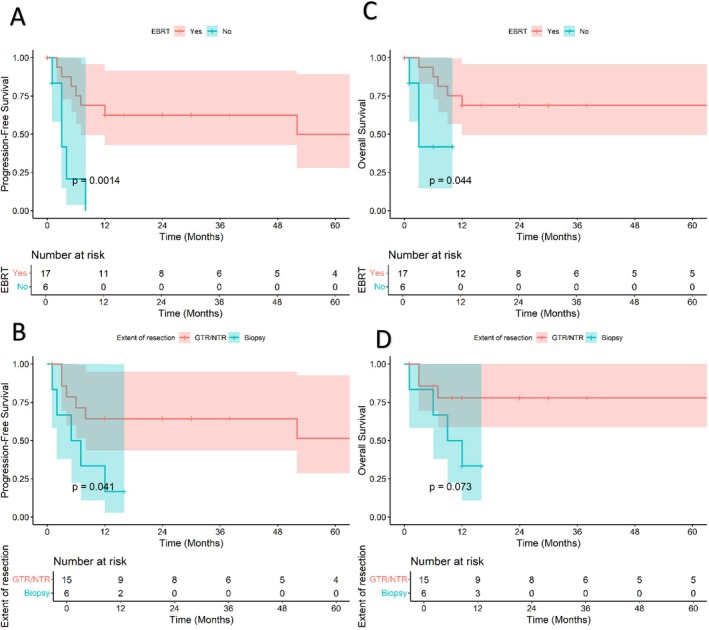
(A) Kaplan Meier curve for progression‐free survival of NF1 high‐grade glioma cohort for EBRT; (B) Kaplan Meier curve for progression‐free survival of NF1 high‐grade glioma cohort for extent of resection; (C) Kaplan Meier curve for overall survival of NF1 high‐grade glioma cohort for EBRT; (D) Kaplan Meier curve for overall survival of NF1 high‐grade glioma cohort for extent of resection.

For high‐grade gliomas, on univariate analysis, patients who received EBRT had better PFS {3‐year: 62.5% (42.8%– 91.4%) vs. NA, *p* = 0.001} and OS {3‐year: 68.7% (49.4%–95.7%) vs. NA, *p* = 0.04}. The extent of resection, biopsy vs. near or gross total resection, was significant for PFS {3‐year: NA vs. 64.2% (43.5%–95%), *p* = 0.04} and showed a trend towards significance for OS {3‐year: NA vs. 77.9% (58.7%–100%), *p* = 0.07}. The results are depicted in Figure 1D. Multivariate analysis of significant predictors with the Cox regression model was precluded by the small number of events and violation of proportional hazards assumptions on testing with scaled Schoenfeld residuals. Analysis of restricted mean survival times failed to elucidate any factors (extent of resection, EBRT, histologic grade) significantly associated with improved survival (Figure S1).

For low‐grade gliomas, on univariate analysis, there was no difference in patients treated with definitive RT vs. chemotherapy with a 3‐year PFS and OS of {100% vs. 89.6% (77%–100%), *p* = 0.23} and (100% vs. 100%, *p* = NA), respectively.

### 3.2 Neurofibromatosis 2

We found 28 patients fulfilling the revised Manchester diagnostic criteria for NF2, of which 26 patients (92.9%) had the presence of bilateral vestibular schwannomas, with or without other tumors. The second most common CNS tumor in patients with NF2 was meningioma, seen in 18 patients (64.3%) after schwannoma. Ependymoma was seen in 7 patients (25.0%). The majority of these patients were young adults (between 19 and 35 years of age) at diagnosis (15 patients, 53.6%), with no gender predilection (M: F = 1:1). Hearing loss and headache were the most common presenting symptoms owing to vestibular schwannomas. Twenty‐one (75%) patients underwent surgery as the definitive modality of treatment, of which 12 (42.8%) patients received adjuvant radiotherapy, 1 (3.5%) patient received adjuvant bevacizumab, 2 (7.1%) patients received upfront radiotherapy and 5 (17.8%) patients were solely kept on observation.

The median follow‐up for the NF2 cohort was 40.5 months (95% CI: 1–199 months), with a 5‐year PFS and OS of 37.3% (95% CI: 20.5%– 68.1%) and 100%, respectively. For patients with schwannoma, a 5‐year PFS of 42.5% (95% CI: 22.7% – 79.5%) and a 5‐year OS of 100% were achieved. For patients with meningioma, a 5‐year PFS of 25% (95% CI: 4.5%–100%) and a 5‐year OS of 100% (95% CI: 0.95–NA) were achieved. For the 2 patients with ependymoma, one had progressed at 39 months but could be salvaged with RT; both are currently on follow‐up and alive with no evidence of disease. For ependymoma, the median PFS was 39 months (95% CI: 0.95–NA), while the median OS was not reached, with a 5‐year OS of 100%.

### 3.3 Von Hippel Lindau

Patients with von Hippel–Lindau (VHL) syndrome exclusively showed haemangioblastoma in the central nervous system, with almost all (12 of 13 patients, 92.3%) occurring in the cerebellum. Three of these patients also suffered from renal cell carcinoma, one patient had an epididymal cyst, and six patients had associated pancreatic and renal cysts. Retinal haemangioblastoma with or without retinal detachment was seen in 5 patients. Most (61.5%) patients were between 19 and 35 years of age. Twelve patients were operated with near‐total resection followed by observation.

The median follow‐up for this cohort was 66.5 months (95% CI: 3–426 months), with 5‐year PFS and OS of 77.92% (95% CI: 54.6%–100%) and 100% respectively.

### 3.4 Tuberous Sclerosis

Clinico‐radiological diagnosis of TSC was established in 6 patients (3 males and 3 females). The most common presenting complaint was seizure. Three patients were diagnosed to have supratentorial SEGA and were offered safe maximal resection followed by observation; one patient received adjuvant mTOR inhibitor (Everolimus). Two patients had CNS tubers and are being observed. One patient was diagnosed with astrocytoma, WHO Grade II, who received adjuvant chemoradiation followed by adjuvant temozolomide and is currently alive with disease.

The median follow‐up was 71 months (95% CI: 1–228), with 5‐year PFS and OS of 75% (95% CI: 42.5%–100%) and 100%, respectively.

For cross‐syndrome interpretability, a summary of survival outcomes across NF1, NF2, VHL, and TSC is presented in Table 4. While statistical comparisons were not performed due to heterogeneity in baseline characteristics and disease biology, descriptive differences in survival outcomes are evident. All patients with VHL and TSC were alive at 5 years, while patients with NF1 and NF2 had relatively more variable outcomes, likely due to differences in tumor grade and multi‐modal treatment.

**TABLE 4:** Descriptive comparison of survival outcomes across syndromes.

Syndrome	*N* (%)	Median follow‐up (months)	PFS (%)	OS (%)
NF1	74 (61.2)	36.5	76.4% (3 years)	87.7% (3 years)
NF2	28 (23.1)	40.5	37.3% (5 years)	100% (5 years)
VHL	13 (10.7)	66.5	77.9% (5 years)	100% (5 years)
TS	6 (5)	71	75% (5 years)	100% (5 years)

Abbreviations: NF, neurofibromatosis; OS, overall survival; PFS, progression free survival; TS, tuberous sclerosis; VHL, Von Hippel Landau Syndrome.

## 4 Discussion

The management of phakomatoses‐associated CNS tumors requires achieving a careful balance between achieving durable survival and disease control versus causing minimal treatment‐related toxicity in patients who are typically young and have to endure the resultant long‐term morbidity. The current publication represents a large single institutional experience of multidisciplinary neuro‐oncologic management in these tumors over a two‐decade span in which definitions, consensus criteria for diagnosis, imaging modalities, and management strategies continued to evolve.

The disease‐related outcomes achieved are gratifying and comparable to similar reported retrospective series [10, 12, 20]. The encouraging 3 year‐OS reported for HGG is supported by limited pre‐existing literature [11] and is mainly driven by the benefit provided by RT in this instance and leads the authors to conclude that the increased radiosensitivity of the normal brain in this setting should not act as a deterrent to providing RT as a life‐extending therapy in these patients in the absence of alternative efficacious therapies.

With regards to NF2‐associated tumors, a notable point pertains to the efficacy of RT in salvaging post‐surgery progression (two of four cases) in NF2‐associated meningioma and schwannoma (3 of 10 cases). The efficacy of RT in providing durable tumor control should always be balanced against the risk of malignant transformation [19]. Additionally, the high incidence of post‐RT inflammation and edema and a resultant increase in tumor size can lead to confusion between progression and pseudo‐progression; indeed, this has been recognized in recently reported large series that have advocated waiting till 5 years before ascribing definitive post‐RT failure [21]. Therefore, one must advocate a strategy of reserving RT for progressive or symptomatic residual disease and avoidance of precipitate interventions with salvage therapies in the event of post‐RT increase in tumor size unless associated with rapid neurological worsening and very obvious progression.

The encouraging survival seen in TS and VHL also lends substance to careful image‐based follow‐up and minimal single modality intervention (surgery, mTORi) in the setting of symptomatic disease. In TSC‐related tumors such as SEGA, the availability of mTOR inhibitors with proven efficacy supports reserving RT for progressive, refractory disease or when systemic therapy is not feasible [22]. Multimodality therapy in these rare syndromes should always be discussed in MDJCs whenever feasible.

The current publication has the inherent advantages of a relatively large sample size and treatment under a relatively consistent treatment paradigm under the auspices of a multidisciplinary neuro‐oncology practice with MDJCs to elucidate and achieve consensus on key treatment decisions. The data are gratifying and will serve as a useful benchmark for planning future studies. The weaknesses of the study pertain to its single institutional origin, retrospective nature, and long duration of data collection (wherein diagnostic criteria for definition have changed over time). A key limitation of our study is the limited availability of molecular or genomic profiling data in the cohort. As many patients were treated in the earlier years of the study period, comprehensive tumor sequencing was not routinely performed. Molecular diagnostics were selectively done in a small subset of patients when clinically indicated or when considered for targeted therapies. Therefore, we could not systematically analyze the impact of specific genetic alterations on prognosis or treatment response. Future prospective studies incorporating routine molecular profiling are warranted to guide targeted therapy selection and to understand the underlying biology of phakomatoses‐associated CNS tumors. In addition, this publication has a predominant population of patients who have not been treated with newly instated treatments in the pertinent disease entities, especially antiangiogenic and targeted therapies such as oral Tyrosine kinase inhibitors.

Several opportunities arise out of the current studies that may significantly improve outcomes in future studies. The constitutive activation of Mitogen‐Activated Protein Kinase (MEK) in NF1 allows for targeting of this pivotal pathway. Successful implementation in NF1‐associated plexiform neurofibroma [8] and the efficacy demonstrated with MEK inhibition in Pediatric Low‐Grade Gliomas (PLGGs) [23] allows for successful extrapolation of this paradigm to NF1‐associated low‐grade gliomas [24]. The daily dosage scheduling for these tumors may prove to be a challenge, which is likely to be addressed by a newer generation of MEK inhibitors that require weekly usage. The typical exclusion of NF patients from these trials, the lack of clarity in the optimal duration of therapy, and the exorbitant cost of treatment represent major challenges in further implementation of these strategies. Similar incremental benefits have been observed in the setting of NF2‐associated vestibular schwannoma via targeting of Vascular Endothelial Growth Factor (VEGF) with Bevacizumab [25] and in all associated tumors, with maximal benefit in meningiomas and non‐vestibular schwannomas via targeting of Focal Adhesion Kinase (FAK) via the usage of Brigatinib (a canonical ALK inhibitor) [6]. The development of these strategies can lead one to envisage a treatment strategy combining these to target various tumor entities throughout the course of the patient's life with judicious usage of radical surgery and highly conformal RT. The overall increased radiosensitivity in NF syndromes renders these patients particularly suitable for particle beam therapy, such as Proton Beam Therapy (PBT), and early results with limited volume datasets appear to be encouraging [26].

The ideal treatment strategy in phakomatoses syndromes would be a systemic agent showing potent activity against all tumor entities in the syndrome. This is challenging to provide in NF (especially NF2) due to the varied presentation of tumors and the wide spectrum of mutations in the underlying genes. Success has been more readily achieved in a setting such as TS, wherein targeting entities such as SEGA with Mammalian Target Of Rapamycin (mTOR) inhibitors such as everolimus [27, 28] has increasingly led to patients being treated preoperatively based on the high index of clinical‐radiological suspicion. Similar potential exists for VHL, wherein the initial efficacy of the Hypoxia Inducible Factor (HIF 2 alpha) inhibitor Belzutifan was demonstrated in VHL, and VHL‐associated renal cell carcinoma (RCC) [29] was successfully extrapolated to cerebellar hemangioblastoma. The challenges to the implementation of these novel therapeutics are several and pertain to cost, exclusion from clinical trials, exclusion from patient support programs due to lack of on‐label indications, lack of sufficient patient advocacy, and a site‐specific paradigm dominating modern oncology wherein such multi‐site diseases may inevitably take the back seat. Focused multidisciplinary clinical and translational research and multi‐centric syndrome‐specific trials may lay the platform in the future to mitigate these drawbacks and improve both the quality and quantity of life in the young and unfortunate victims of these uncommon disease entities.

The findings of this study also carry important implications for neuro‐oncology practice in resource‐limited settings. In many LMICs, access to advanced molecular diagnostics, targeted therapies, and proton therapy remains absent, limited, or cost‐prohibitive. Our results show that favorable survival outcomes can yet be achieved using a practical and risk‐adapted approach: maximal safe resection, carefully selected use of conformal radiotherapy, and widely available systemic agents such as vinblastine and temozolomide. Also, multidisciplinary joint clinics (MDJCs) consensus‐based decisions ensure rational allocation and sequencing of available resources. While molecular profiling and targeted therapies will surely improve precision, this dataset provides a benchmark for what is realistically achievable in current practice using conventional modalities.

## 5 Conclusion

Multidisciplinary collaboration is essential in the management of phakomatoses‐associated brain tumors. Much progress is pending in the development of novel therapeutics and their evaluation in clinical trials.

## Author Contributions

**Anuradha Krishnan:** data curation (equal), formal analysis (equal), investigation (equal), methodology (equal), validation (equal), visualization (equal), writing – original draft (equal), writing – review and editing (equal). **Yamini Baviskar:** data curation (equal), investigation (equal), methodology (equal), writing – original draft (equal). **Abhishek Chatterjee:** conceptualization (equal), data curation (equal), formal analysis (equal), investigation (equal), methodology (equal), project administration (equal), supervision (equal), validation (equal), visualization (equal), writing – original draft (equal), writing – review and editing (equal). **Archya Dasgupta:** resources (equal). **Sridhar Epari:** resources (equal). **Arpita Sahu:** resources (equal). **Girish Chinnaswamy:** resources (equal). **Nandini Menon:** resources (equal). **Aliasgar Moiyadi:** resources (equal). **Tejpal Gupta:** resources (equal). **Jayant Sastri‐Goda:** conceptualization (equal), data curation (equal), formal analysis (equal), investigation (equal), methodology (equal), project administration (equal), supervision (equal), validation (equal), visualization (equal), writing – original draft (equal), writing – review and editing (equal).

## Funding

The authors have nothing to report.

## Conflicts of Interest

The authors declare no conflicts of interest.

## Supporting information


**Figure S1:** Restricted mean survival time (RMST) and restricted mean time lost (RMTL) for overall survival in the NF1 high grade glioma cohort for EBRT.

## Data Availability

Research data are not shared.
