# Supplementary material for: Presentation and Outcomes of CNS Tumors Associated With Phakomatoses Syndromes From a Specialized Neuro‐Oncology Practice in India
Source: Cancer Med. 2026 Feb 4;15(2):e71483. doi: 10.1002/cam4.71483 (PMC12873450; doi:10.1002/cam4.71483)

Supplementary figure 1– Restricted mean survival time (RMST) and restricted mean time lost (RMTL) for overall survival in the NF1 high grade glioma cohort for EBRT


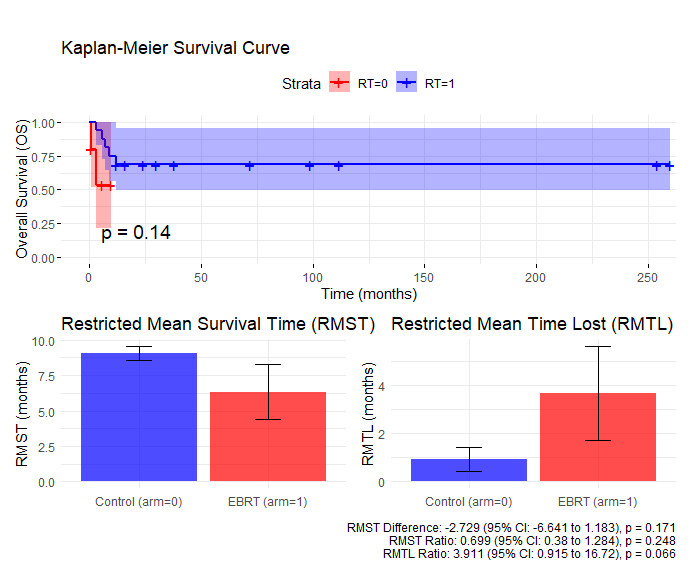

Supplement: Supplementary file 1 — Figure S1: Restricted mean survival time (RMST) and restricted mean time lost (RMTL) for overall survival in the NF1 high grade glioma cohort for EBRT. [file CAM4-15-e71483-s001.docx]
